# Physicochemical, Mechanical and Biological Characterization of a PCL/PLA-Based Biocomposite for Potential Dental Post Applications in Primary Teeth

**DOI:** 10.3390/polym18182258

**Published:** 2026-09-16

**Authors:** Candy del Carmen Gamboa-Solana, David Alejandro Aguilar-Pérez, Marco Antonio Álvarez-Pérez, Víctor Ermilo Arana-Argáez, Rossana Faride Vargas-Coronado, Alexis Bernardo Sabido-Barahona, Beatriz Adriana Rodas-Junco, Martha Gabriela Chuc-Gamboa, Juan Valerio Cauich-Rodríguez

**Affiliations:** 1 Facultad de Odontología, Universidad Autónoma de Yucatán, Calle 61 A #492 A x 90 y Av. Itzáes, Centro, Mérida 97000, Yucatán, Mexico; a18018519@uady.alumnos.mx (C.d.C.G.-S.);; 2 Laboratorio de Bioingeniería de Tejidos, División de Estudios de Posgrado e Investigación, Facultad de Odontología, Universidad Nacional Autónoma de México, Coyoacán, Ciudad de México 04510, Mexico; 3 Laboratorio de Farmacología, Facultad de Química, Universidad Autónoma de Yucatán, Mérida 24085, Yucatán, Mexico; 4 Centro de Investigación Científica de Yucatán, Unidad de Materiales, Calle 43 No. 130 x 32 y 34, Colonia Chuburná de Hidalgo, Mérida 97205, Yucatán, Mexico; 5 Laboratorio de Células Troncales, Facultad de Odontología, Universidad Autónoma de Yucatán, Calle 61 A #492 A x 90 y Av. Itzáes, Centro, Mérida 97000, Yucatán, Mexico; 6 SECIHTI—Facultad de Ingeniería Química, Universidad Autónoma de Yucatán, Periférico Norte Kilómetro 33.5, Tablaje Catastral 13615, Chuburná de Hidalgo Inn., Mérida 97203, Yucatán, Mexico

**Keywords:** polycaprolactone, polylactic acid, biocomposite, dental post, primary teeth

## Abstract

Early childhood caries frequently affects maxillary anterior primary teeth, and their rehabilitation remains a clinical challenge due to the lack of suitable resorbable intracanal posts. This study aimed to develop and characterize a biodegradable polymer-ceramic biomaterial for potential application in primary tooth rehabilitation. Polycaprolactone (PCL) and polylactic acid (PLA) composite filaments (50:50), reinforced with 20 wt.% α-tricalcium phosphate and 5 wt.% zirconia, were produced using two different processing routes involving a Noztek Touch and a Brabender Plasti-Corder extruder. The developed biocomposites obtained by single extrusion with a Brabender Plasti-Corder exhibited good ceramic distribution in the polymeric matrix but exhibited lower mean compressive modulus values (155.98 MPa vs. 258.44 MPa), which were not statistically significant. The composite biomaterial showed degradation under alkaline conditions, whereas only limited mass loss was observed under acidic conditions and in hydrogen peroxide, with minimal mass loss in distilled water. These composites showed favorable biocompatibility with dental pulp stem cells, although no antimicrobial activity was observed. Furthermore, the incorporation of zirconia may contribute to radiopacity for radiographic monitoring. These findings support the potential of the developed composite biomaterial as a candidate biodegradable intracanal post for pediatric restorative dentistry applications.

## 1. Introduction

Dental caries is one of the most prevalent chronic diseases in childhood and remains a major public health problem worldwide. In children under six years of age, this condition is defined as early childhood caries (ECC), which predominantly involves the maxillary primary incisors. In addition to dental caries, traumatic injuries are also common in this age group and may result in extensive loss of coronal tooth structure. Both conditions negatively affect children’s quality of life and may lead to functional, esthetic, psychological, and systemic consequences [1,2,3].

In advanced stages, extensive crown destruction compromises the retention of conventional restorations. Although extraction was once considered the treatment of choice for severely damaged primary teeth, current pediatric dentistry aims to preserve these teeth until their physiological exfoliation whenever possible. Therefore, pulpectomy followed by intracanal retention with a dental post and final coronal restoration has become a widely accepted treatment option for restoring teeth with extensive structural loss [3,4].

An ideal dental post for primary teeth should be resorbable, provide sufficient strength to support the core, exhibit adequate compressive strength and a good distribution of forces affecting the root, and respect the eruption process of the permanent tooth, while providing adequate support for the coronal restoration [5,6]. Despite these requirements, no commercially available resorbable post currently exists.

Several types of dental posts have been proposed for restoring severely damaged primary teeth. These include prefabricated systems, such as glass fiber-reinforced posts, ceramic and glass fiber posts, or custom-made posts, including alpha-, delta-, and omega-shaped metals, composite, and biological posts [7]. Biological posts can undergo physiological root resorption; however, their clinical application is limited by the need for donor teeth, complex preparation procedures, sterilization requirements, and low parental acceptance [5]. Consequently, glass fiber posts remain one of the most used options for intracanal retention in the restoration of primary teeth. Nevertheless, these posts are designed for permanent teeth and therefore require clinical modification before being adapted for use in primary teeth [8]. Furthermore, currently available post systems do not provide dimensions specifically designed for primary dentition or accommodate physiological root resorption.

To overcome these limitations, recent studies have explored the development of biodegradable intracanal posts based on polymeric materials. Xu et al. evaluated resorbable PLA intracanal posts and demonstrated clinical performance comparable to that of glass fiber posts, with improved gingival health and fewer postoperative complications [9]. More recently, Wang et al. fabricated biodegradable posts with a geometry comparable to conventional fiber posts using a PLA/PCL matrix reinforced with amorphous calcium phosphate (ACP) using injection molding and evaluated their mechanical properties and in vitro biocompatibility [10]. These studies demonstrate the growing interest in biodegradable intracanal posts and support the continued investigation of polymer-based systems for this application. However, the available literature on biodegradable dental posts for primary teeth remains limited, particularly regarding polymer-ceramic systems. Recent studies have investigated PCL and PLA/PCL matrices incorporating calcium phosphate phases and fabricated by additive manufacturing, highlighting the influence of ceramic incorporation on morphology, porosity, degradation, and mechanical properties [11,12]. Nevertheless, these studies have focused predominantly on tissue-engineering scaffolds rather than intracanal posts for primary teeth. Thus, although biodegradable PLA-based and PLA/PCL-based intracanal posts have been investigated, the incorporation of α-TCP and ZrO_2_ into the PCL/PLA matrix for resorbable intracanal posts in primary teeth has not been widely reported. Consequently, the influence of different processing routes on morphology, filler distribution, porosity, thermal behavior, and mechanical performance of polymer-ceramic composites for this application remains insufficiently investigated.

Polycaprolactone (PCL) and polylactic acid (PLA) have attracted interest in biomedical and dental applications due to their biocompatibility, biodegradability, and relatively low cost [13,14]. Furthermore, blending PCL with PLA combines the flexibility and slow degradation rate of PCL with the higher stiffness and mechanical strength of PLA, resulting in a material with more balanced mechanical properties and a more controlled degradation profile [15,16,17]. To further reinforce the polymer matrix, ceramic particles were incorporated. Alpha-tricalcium phosphate (α-TCP) was incorporated because of its biocompatibility, osteoconductive properties, and similarity to the mineral composition of dental tissues [18,19]. Recent studies have also investigated calcium phosphate-reinforced PCL composites fabricated by additive manufacturing, highlighting the potential of these materials to combine biodegradable polymer matrices with bioactive ceramic phases [11]. Zirconia (ZrO_2_) particles were also incorporated as reinforcing fillers because of their radiopacity and potential antimicrobial properties [20,21]. The combination of these components therefore provides an opportunity to integrate the complementary characteristics of a biodegradable polymer matrix with calcium phosphate and zirconia ceramic phases in a single biomaterial for dental applications.

Therefore, the aim of this study was to evaluate the physicochemical and mechanical properties, cell viability, and antimicrobial activity of two biomaterials with identical chemical composition consisting of PCL, PLA, α-TCP, and ZrO_2_. The biomaterials were processed using two different processing routes, the Noztek Touch and Brabender Plasti-Corder systems, to compare the resulting filament morphology, filler distribution, porosity, thermal behavior, and mechanical performance.

## 2. Materials and Methods

### 2.1. Materials

Polycaprolactone pellets (molecular weight 80,000 g/mol) were acquired from Sigma-Aldrich (St. Louis, MO, USA), while commercial polylactic acid in filament form was purchased from Steren (México City, México). Alpha Tricalcium Phosphate was synthesized according to the method described by Fernández et al. [22] and provided by the Macromolecular Chemistry Laboratory (CICY, Mérida, México). Zirconium oxide with a particle size of 5 μm (molecular weight 123.22 g/mol) was acquired from Sigma-Aldrich (St. Lous, MO, USA).

For cytotoxicity assays, Dulbecco’s Modified Eagle Medium (DMEM) from Biowest^®^ (Nuaillé, France) was supplemented with 10% fetal bovine serum (FBS), penicillin (100 UI/mL), streptomycin (100 μg/mL), and L-glutamine. Cell viability was evaluated using an MTT Assay Kit (ab211091, Abcam, Cambridge, UK). The cells were obtained after the patient and his legal guardian provided signed informed consent in accordance with approval from the Research Ethics Committee of the Autonomous University of Yucatán (UADY) under approval number CIE-06-2017. Fluorescence analysis was performed using CellTracker™ Green CMFDA (Thermo Fisher Scientific, Waltham, MA, USA) and observed with epifluorescence microscopy.

### 2.2. Preparation of Biocomposites

#### 2.2.1. Preparation of Composite Filaments Using a Noztek Touch

**Figure 1:**
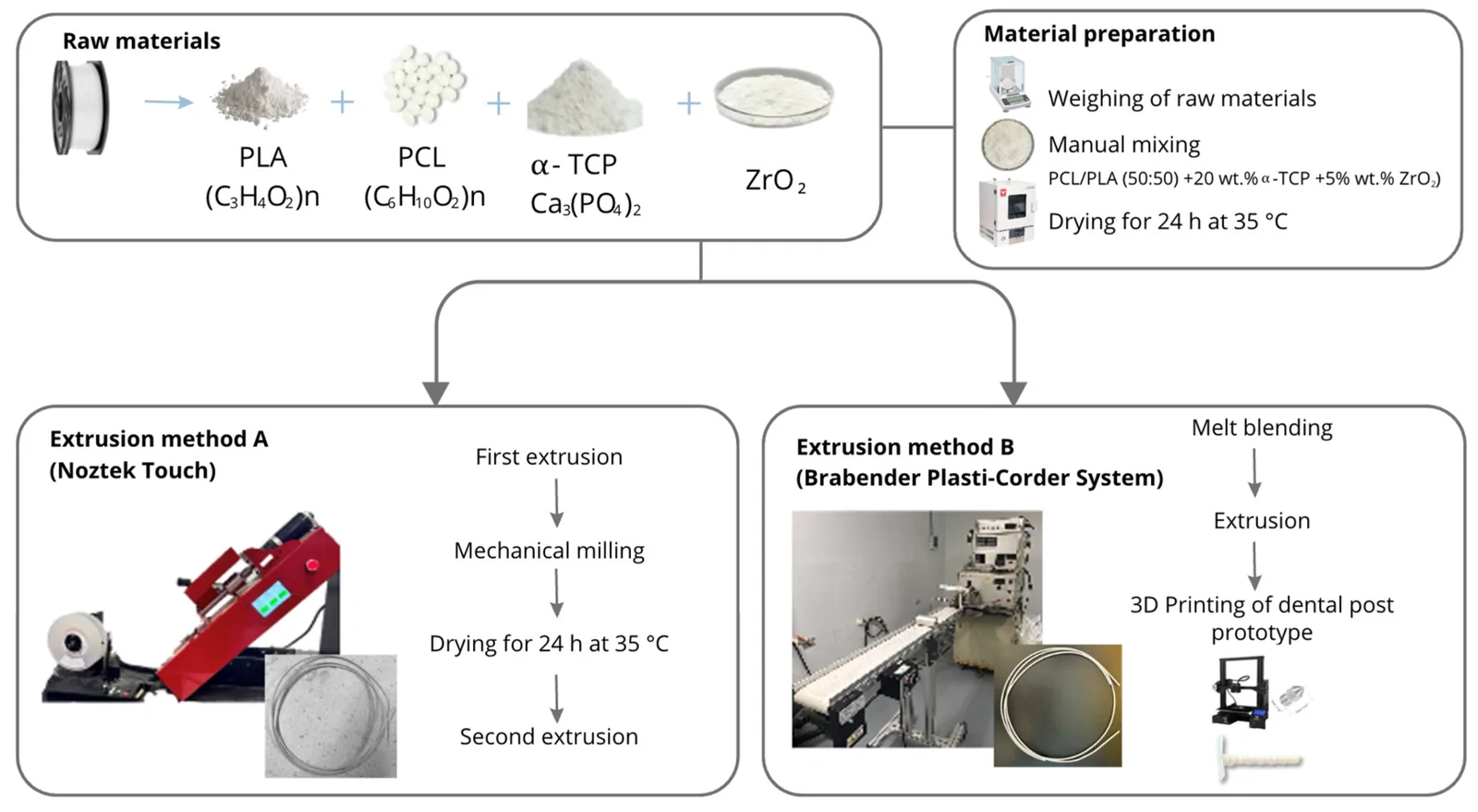
Workflow for the preparation of the PLA/PCL/α-TCP/ZrO_2_ composite biomaterials, including raw material preparation, drying, extrusion using different processing systems, and fabrication of the dental post prototype by 3D printing.

The composite biomaterials were prepared from a 50:50 PCL/PLA blend containing 20 wt.% α-TCP and 5 wt.% ZrO_2_, based on the total composite mass (Table 1). These compositions were selected based on the compressive behavior of preliminary formulations with up to 30 wt.% of α-TCP and based on the radiopacity and potential antimicrobial effect of 1 wt.% and 5 wt.% of ZrO_2_ formulations. PLA filament was ground using a Brabender mill fitted with a No. 2 sieve. The α-TCP and ZrO_2_ powders were homogenized in an agate mortar to minimize particle agglomeration. The required amounts of each component were weighed and manually premixed in glass Petri dishes. Prior to extrusion, the mixtures were dried in a vacuum oven at 35 °C for 24 h to remove residual moisture. Figure 1 presents a schematic overview of the preparation process.

Subsequently, the mixture was processed using a Noztek Touch single-screw extruder (Noztek, West Sussex, UK) equipped with a 1.75 mm nozzle (method A). Extrusion was performed at 170 °C and a screw speed of 8 rpm. The resulting filament was reground and re-extruded under the same processing conditions as an additional processing step intended to promote material homogeneity and ceramic filler dispersion throughout the polymer matrix.

#### 2.2.2. Preparation of Composite Filaments Using a Brabender Plasti-Corder System

Following the same powder preparation, blending, and drying procedures described for the Noztek Touch process, the composite formulation was processed using a different processing route that included a preliminary melt-compounding step. The composite was first melt-compounded using a Brabender Plasti-Corder internal mixer (Brabender GmbH and Co. KG, Duisburg, Germany) at 170 °C and a rotor speed of 20 rpm. This preliminary melt-compounding step was performed to improve the dispersion of the ceramic fillers within the polymer matrix to obtain a homogeneous composite. The resulting material was subsequently ground in a Brabender mill and extruded using a Brabender Plasti-Corder single-screw extruder equipped with a ½-inch screw (L/D = 25:1) (method B). The temperatures of the three-barrel heating zones and the die were maintained at 170 °C, and the screw speed was set at 11 rpm to produce composite filaments suitable for subsequent 3D printing. Figure 1 presents a schematic overview of the preparation process.

Different batch sizes were prepared according to the processing capacity of each extrusion system while maintaining the same relative composition. Although the composite formulations underwent the same initial powder preparation, blending, and drying procedures, the subsequent processing routes differed. Therefore, the comparison between the resulting filaments represents an evaluation of two distinct processing routes rather than an isolated assessment of the extruder type.

### 2.3. Composition and Structural Characterization of Composite Filament Extruded by Noztek Touch and Brabender Plasti-Corder System

#### 2.3.1. Fourier Transform Infrared Spectroscopy (FTIR)

FTIR spectra were acquired using a Nicolet Thermo-Scientific 8700 FTIR spectrometer (Thermo Fisher Scientific, Madison, WI, USA) equipped with an attenuated total reflectance (ATR) accessory. Spectra were recorded in the range of 4000 to 650 cm^−1^, with a resolution of 0.4 cm^−1^, averaging 200 scans per sample. Atmospheric compensation for H_2_O and CO_2_ was applied prior to analysis.

#### 2.3.2. Raman Spectroscopy

Raman spectra were acquired using an inVia™ Raman microscope (Renishaw, plc, Gloucestershire, UK) equipped with a 633 nm He-Ne laser (17 nW), an 1800 lines mm^−1^ diffraction grating, and a 50× objective lens. Spectra were collected over the spectral range of 3200–100 cm^−1^ with an acquisition time of 10 s per spectrum.

#### 2.3.3. Thermogravimetric Analysis (TGA)

Thermal characterization of biomaterials was performed using a TGA 5500 (TA Instruments, New Castle, DE, USA), over a temperature range of 30 °C to 500 °C, at a heating rate of 10 °C/min, under a nitrogen atmosphere. The initial sample mass was approximately 8–12 mg, depending on sample availability.

### 2.4. Surface Properties of Filaments

#### 2.4.1. X-Ray Photoelectron Spectroscopy (XPS)

X-ray photoelectron spectroscopy (XPS) analysis was performed to evaluate the surface elemental composition of the composite filaments using a Thermo Scientific K-Alpha X-ray Photoelectron spectrometer (Waltham, MA, USA), equipped with a monochromatic Al Kα X-ray source (1486.6 eV). Survey spectra were acquired over the energy range of 0 to 1200 eV to identify the elements present on the surface of each sample. Additionally, high-resolution spectra were collected for C 1s, O 1s, Ca 2p, P 2p, and Zr 3d.

#### 2.4.2. Scanning Electron Microscopy

The composite filaments were cryo-fractured under liquid nitrogen conditions to expose the internal structure. The morphology of the composite biomaterial was examined by scanning electron microscopy (SEM) using a JSM-6360LV microscope (JEOL Ltd., Akishima, Tokyo, Japan). Prior to imaging, the specimens were sputter-coated with a thin layer of gold using a Desk II Denton Vacuum sputter coater (Moorestown, NJ, USA) for 50 s. SEM images were acquired using an accelerating voltage of 20 kV.

### 2.5. Mechanical Properties Under Compression

A compressive test was conducted to evaluate the compressive modulus of the PCL/PLA/α-TCP 20%/ZrO_2_ 5% composite biomaterial. Cylindrical specimens measuring approximately 2.5 mm in height and 1.55 mm in diameter were analyzed using a MiniShimadzu AGS-X universal testing machine equipped with a 1000 N load cell. Compression was applied at a crosshead speed of 0.5 mm/min, adapted from ASTM D695 [23].

Five specimens were evaluated for each processing route (n = 5). Statistical analysis was performed using Minitab statistical software version 22.5.1 (Minitab, LLC, State College, PA, USA). Data normality was assessed using the Shapiro–Wilk test, and the compressive modulus values between the Noztek Touch and Brabender Plasti-Corder groups were compared using a two-sample Student’s *t*-test. Statistical significance was set at *p* < 0.05. Results are reported as mean ± standard deviation.

### 2.6. Accelerated Degradation

The accelerated degradation behavior of the individual components (PCL, PLA, α-TCP, and ZrO_2_) and the composite biomaterials was evaluated using three specimens per group. The samples were immersed in four aqueous media: distilled water, 30% H_2_O_2_, 2N HCl, and 5N NaOH under reflux conditions at 100 °C for 8 h. After degradation, the specimens were rinsed with distilled water, dried at room temperature, and subsequently characterized by FTIR and Raman spectroscopy. The resulting spectra were compared with those of the corresponding non-degraded materials to identify chemical and structural changes induced by the degradation treatments.

### 2.7. Biological Studies

#### 2.7.1. Cytotoxicity Test

Cell viability was evaluated using 3-(4,5-dimethylthiazol-2-yl)-2,5-diphenyl tetrazolium bromide (MTT) with human dental pulp stem cells (hDPSCs) cultured in 96-well plates at a density of 1 × 10^4^ cells per well. The assay included a negative control group (cells cultured in extract-free medium) and a test group. After 24 h of incubation at 37 °C with 5% CO_2_, MTT solution was added and incubated for an additional 3 h. Formazan crystals were dissolved using dimethyl sulfoxide (DMSO), producing a purple solution. Absorbance was measured at 492 nm.

The MTT assay was performed on two independent experimental occasions (biological replicates). In each experiment, five wells per material were analyzed as technical replicates, resulting in a total of 10 measurements per material. The results were expressed as mean ± standard deviation. Statistical analysis was performed using one-way analysis of variance (ANOVA), followed by Tukey’s multiple-comparison test, considering the 10 measurements per group.

#### 2.7.2. Evaluation of Cell Adhesion by CellTracker^TM^ Green CMFDA

To evaluate cell adhesion and distribution on the biomaterial surfaces, hDPSCs were labeled with CellTracker^TM^ Green CMFDA. Cells were cultured in T-75 culture flasks containing Dulbecco’s Modified Eagle Medium (DMEM) supplemented with 10% fetal bovine serum (FBS), penicillin (100 IU/mL^−1^), streptomycin (100 μg/mL^−1^), and L-glutamine at 37 °C in a humidified atmosphere containing 95% air and 5% CO_2_. Cells were incubated with CellTracker^TM^ Green CMFDA in serum and phenol red-free culture medium for 1 h according to the manufacturer’s instructions. After staining, the medium was removed, and the cells were washed with phosphate-buffered saline (PBS) Sigma-Aldrich, St. Louis, MO, USA) before incubation in complete culture medium for an additional 1 h.

Subsequently, the labeled cells were detached by trypsinization and seeded onto sterile composite specimens at a density of 2.5 × 10^4^ cells per sample. Three independent specimens per treatment group were analyzed after 24 h of culture. The samples were washed twice with PBS to remove non-adherent cells and fixed with 2% glutaraldehyde for 15 min at room temperature. Following fixation, the specimens were rinsed with Milli-Q water, and cell adhesion and distribution were examined using an LED fluorescence microscope equipped with an digital camera (AmScope, Irvine, CA, USA) at 10× and 20× magnifications.

In addition, SEM analysis was performed on biomaterial specimens cultured with human dental pulp mesenchymal stem cells to qualitatively evaluate cell attachment and morphology on the material surface. The specimens were prepared using the same gold sputter-coating procedure and analyzed at ×1500 magnification with an accelerating voltage of 8 kV.

#### 2.7.3. Antimicrobial Activity Assays

The antibacterial activity of the biomaterials was evaluated using an agar diffusion assay against *Staphylococcus aureus* ATCC 25923 and *Escherichia coli* ATCC 25922 (American Type Culture Collection [ATCC], Manassas, VA, USA) as representative Gram-positive and Gram-negative bacterial strains, respectively. The assay was performed using three independent specimens per condition. Both bacterial strains were reactivated in nutrient broth at 37 °C for 24 h. The bacterial suspensions were adjusted to a 0.5 McFarland standard (approximately 1.5 × 10^8^ CFU mL^−1^) using sterile saline solution. Then, 100 μL of each bacterial suspension was inoculated onto Mueller Hinton agar plates, and the microorganisms were diffused in the medium using a glass loop. Later, disks 1.5 mm in diameter were cut and placed in Petri dishes containing the Mueller-Hinton culture medium previously inoculated with the microorganisms and then were incubated at 35 °C for 16–18 h.

Cefoxitin was used as a positive control for *Staphylococcus aureus*, whereas amikacin was used as a positive control for *Escherichia coli.* Sterile paper disks without antimicrobial agents were used for both as a negative control.

### 2.8. Prototype of Dental Post by 3D Printing

A preliminary dental post prototype was fabricated using additive manufacturing by fused deposition modeling (FDM). The dimensions of a commercially available glass fiber post (ParaPost Fiber Lux, Coltene) were used as a reference for the design. A three-dimensional computer-aided design (CAD) model was created based on these dimensions and exported as an STL file for printing. The prototype was fabricated using a Creality Ender-3 S1 printer (Shenzhen Creality 3D Technology Co., Shenzhen, China) equipped with a 0.4 mm nozzle. The extrusion temperature was set at 180 °C, while the bed platform temperature was maintained at 60 °C. The additional 3D printing parameters were as follows: layer height, 0.2 mm, and printing speed, 30 mm/s. The dental posts were printed in a vertical orientation using spiralized mode, without internal infill (0% infill), and with 100% concentric wall orientation.

As a preliminary mechanical assessment, small specimens were obtained by sectioning the thicker portions of the 3D-printed dental posts. The specimens were approximately 2.55 mm in length, with a maximum diameter of approximately 1.50 mm, reflecting the tapered geometry of the printed posts. Five specimens were evaluated using the compression testing procedure described in Section 2.5.

## 3. Results and Discussion

### 3.1. Physicochemical and Structural Characterization of Composite Filaments

#### 3.1.1. FTIR Spectroscopy

**Figure 2:**
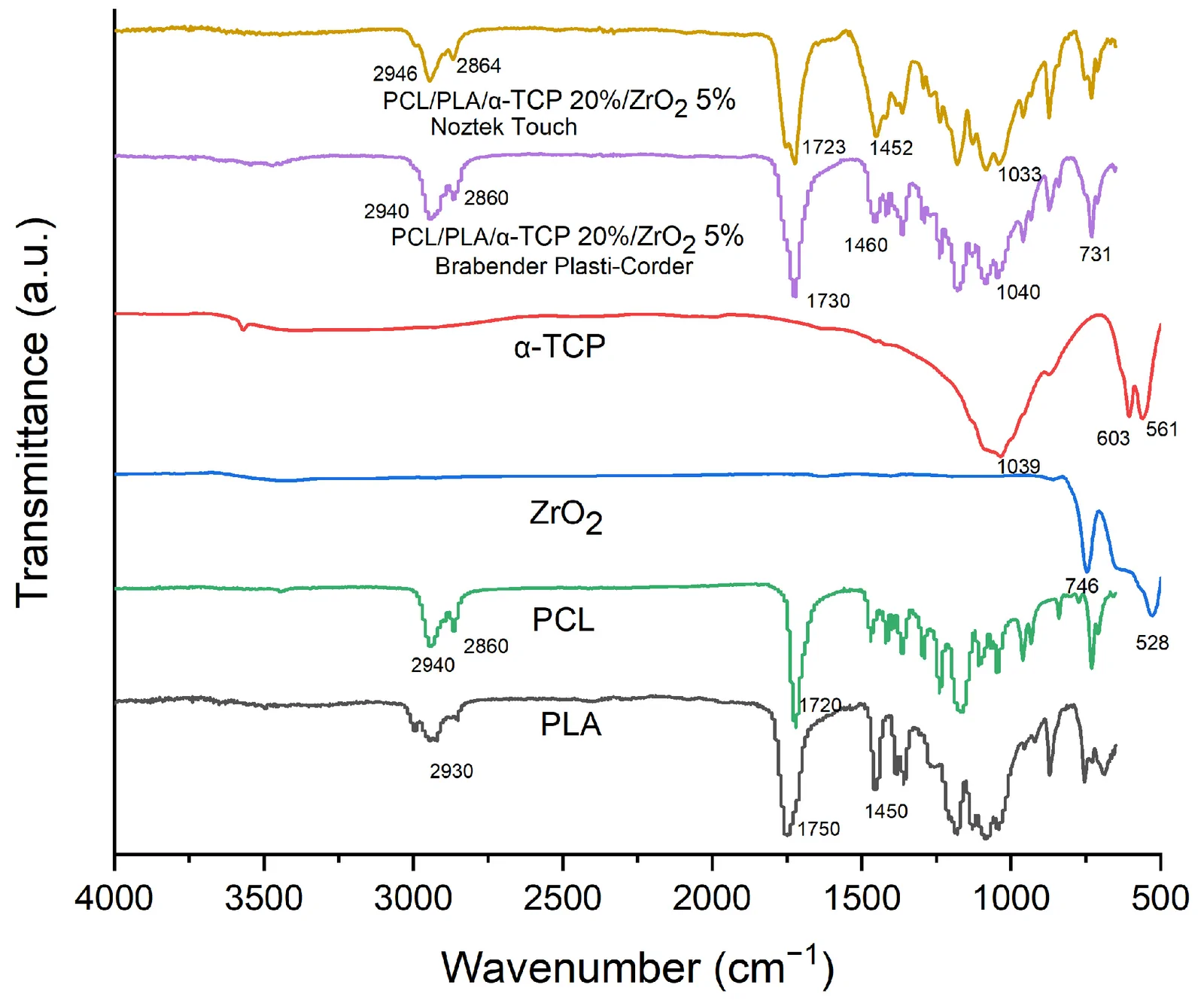
FTIR spectra of individual components and composite biomaterial filaments (PCL/PLA/α-TCP 20%/ZrO_2_ 5%) processed by Brabender Plasti-Corder and Noztek Touch extrusion.

Figure 2 shows the FTIR spectra of the individual components (PLA, PCL, α-TCP, and ZrO_2_) and the PLA/PCL/α-TCP 20 wt.%/ZrO_2_ 5 wt.% composite filaments obtained by two different extruders. PCL and PLA exhibit characteristic absorption bands at approximately 2940 cm^−1^ and 2860 cm^−1^, corresponding to the asymmetric and symmetric stretching vibrations of CH_2_ groups. In addition, both polymers showed a characteristic carbonyl (C=O) stretching band between 1720 cm^−1^ and 1750 cm^−1^ [24].

The FTIR spectrum of α-TCP showed characteristic bands at 1039 cm^−1^ assigned to stretching triply degenerate (ν3) and at 603 cm^−1^ and 561 cm^−1^, corresponding to the anti-symmetric bending vibration of the phosphate group (P–O bending triply degenerate, v4) [18]. The FTIR spectrum of ZrO_2_ exhibits characteristic absorption bands at 746 cm^−1^ and 528 cm^−1^. These bands are associated with Zr–O vibrational modes and are consistent with metal-oxygen vibrations commonly reported for zirconia in the 400–800 cm^−1^ region [25].

For the composite biomaterial filaments fabricated using the Noztek Touch and Brabender Plasti-Corder extrusion systems, the characteristic absorption bands of all constituent materials were preserved. No additional absorption bands or significant spectral shifts were observed after extrusion. Comparison of the spectra obtained from both processing methods revealed no appreciable differences, indicating that the extrusion technique did not alter the chemical structure of the composite biomaterial. The preservation of the characteristic absorption bands further suggests that the composite remained chemically stable during processing, with no evidence of polymer degradation or the formation of new functional groups.

#### 3.1.2. Raman Spectroscopy

**Figure 3:**
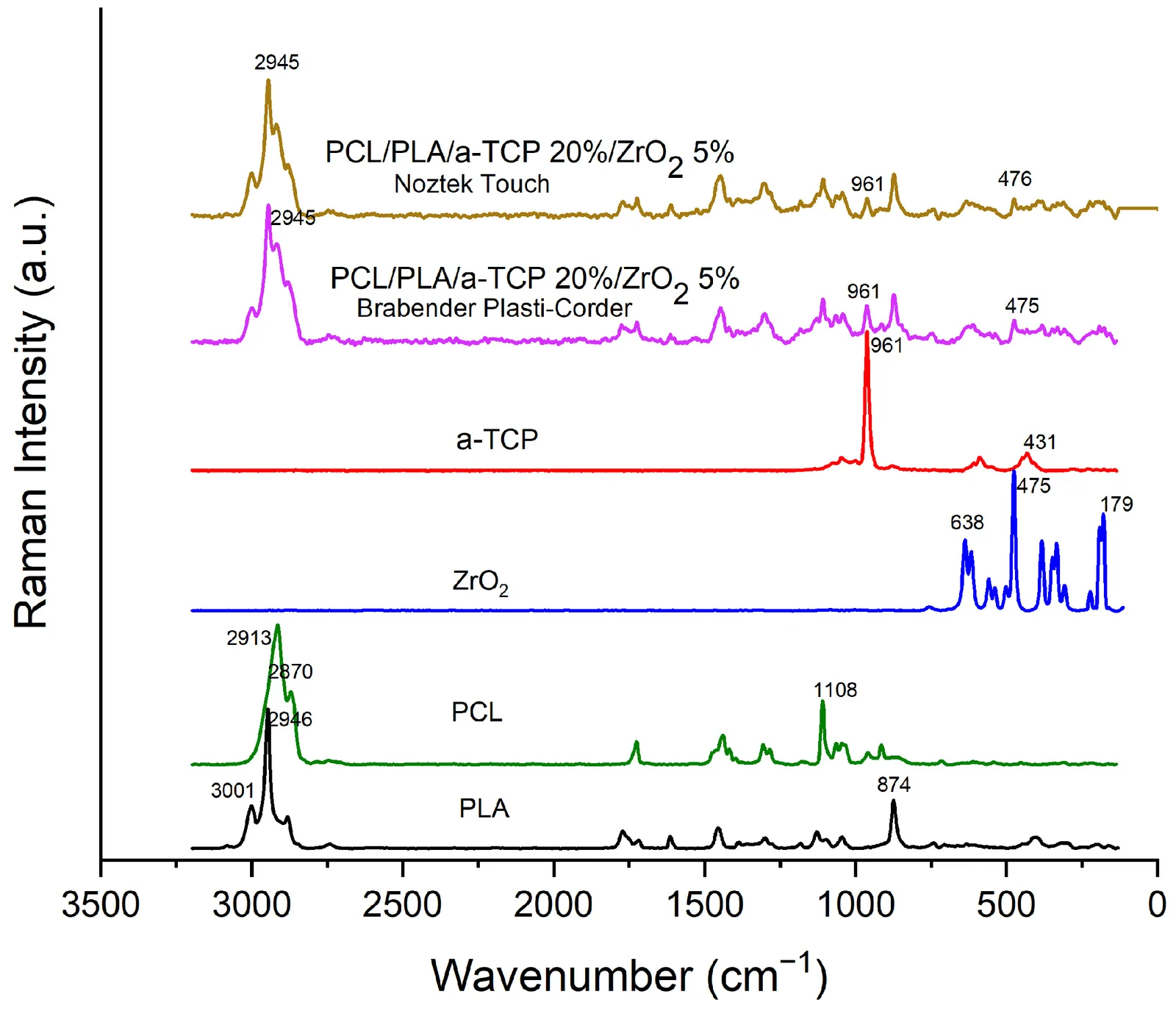
Raman spectra of individual components and composite biomaterial filaments (PCL/PLA/α-TCP 20%/ZrO_2_ 5%) processed by Brabender Plasti-Corder and Noztek Touch extrusion.

The Raman spectrum of the individual components and composite biomaterial filaments was evaluated and displayed in Figure 3. Peaks at 3001 cm^−1^, 2946 cm^−1^, 2913 cm^−1^, and 2870 cm^−1^ were observed, corresponding to C-H stretching vibrations of aliphatic CH_2_ and CH_3_ characteristic of polylactic acid and polycaprolactone [26,27]. In the composite material, the characteristic peak at 2945 cm^−1^ was preserved, and no significant peak shifts were observed, suggesting that the extrusion processes did not significantly alter the chemical structure of the polymeric matrix.

The Raman spectrum of α-TCP showed a characteristic band at 961 cm^−1^, assigned to the ν1 symmetric stretching of PO_4_ groups. This characteristic phosphate band was also identified in both composite materials, confirming the presence of the ceramic phase after processing [18].

For ZrO_2_, characteristic peaks were observed at 638 cm^−1^, 475 cm^−1^, and 179 cm^−1^, corresponding to crystalline lattice vibrations of zirconia. These results confirm the successful incorporation and preservation of ZrO_2_ within the composite biomaterial [28].

#### 3.1.3. Thermogravimetric Analysis (TGA)

**Figure 4:**
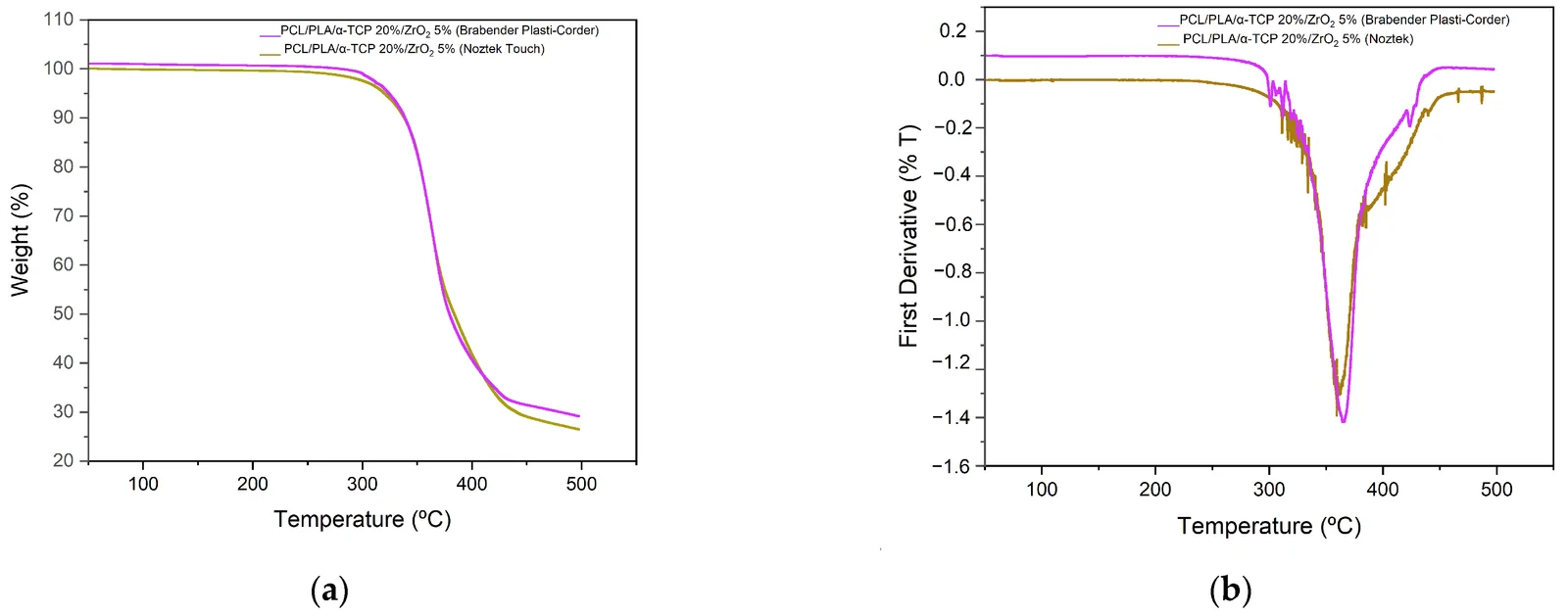
TGA (**a**) and DTGA (**b**) thermograms of filaments (PCL/PLA/α-TCP 20%/ZrO_2_ 5%) extruded with Noztek and the Brabender Plasti-Corder System.

The TGA and DTG curves of PCL/PLA/α-TCP 20%/ZrO_2_ 5% composite biomaterial filaments are shown in Figure 4. The main degradation process began at approximately 300 °C and reached its maximum degradation rate near 362 °C, corresponding to the thermal decomposition of the polymeric matrix. The thermal behavior observed in the present study is consistent with previous reports on PLA/PCL-based systems [29,30,31]. The maximum degradation rate was observed near 362 °C to 366 °C, corresponding to the degradation of the PLA/PCL blend.

A residual mass of approximately 26 wt.% for the Noztek composite biomaterial and 20 wt.% for the Brabender composite biomaterial remained at the end of the analysis. This residue is attributed primarily to the thermally stable inorganic phases, α-TCP and ZrO_2_, which remain after the degradation of the polymeric matrix. The residual mass obtained for the Noztek composite is close to the nominal inorganic content of the formulation (25 wt.%). However, non-zero residual masses have been reported for neat PLA and PCL under certain TGA conditions [32], indicating that the polymeric matrix may also contribute to the measured residual mass. In contrast, the lower residual mass observed for the Brabender composite may be associated with processing-related differences in filler incorporation and distribution and possible filler losses during processing. Therefore, the TGA results alone do not provide sufficient evidence to establish differences in the actual inorganic content of the two composites.

### 3.2. Surface Properties of Filaments

#### 3.2.1. Elemental Composition by XPS

The analysis revealed the presence of carbon, oxygen, and calcium as the main surface elements in the analyzed composites (see Table 2). Carbon and oxygen were attributed to the PLA/PCL polymeric matrix, whereas the calcium was associated with the α-TCP phase. A higher calcium signal was detected in the Brabender composite filament compared with the Noztek composite filament. Since both materials had the same composition, this difference is more likely related to variations in the surface concentration of the TCP particles resulting from the processing method rather than differences in the bulk composition.

Although phosphorus and zirconium were not consistently detected in the survey spectra, high-resolution scans showed weak contributions in the P 2p and Zr 3d regions, located at approximately 134 eV and 184 eV, respectively. The limited detection of these elements in the survey analysis may be related to the surface-sensitive nature of XPS, which probes only the outermost nanometers of the material. Additionally, partial coverage of the ceramic particles by the polymeric matrix may reduce the intensity of inorganic signals due to limited exposure of the ceramic phase at the surface. Similar observations have been reported for polymer-ceramic composites, in which ceramic particles are partially covered by an organic surface layer, limiting the detection of inorganic elements by XPS [33].

These findings are consistent with the FTIR, Raman, and TGA results, which confirmed the incorporation of α-TCP and ZrO_2_ into the composite biomaterials. Although both extrusion systems produced chemically comparable composite biomaterials, XPS suggests that differences in the processing route may influence the surface distribution of the ceramic fillers.

#### 3.2.2. Surface Morphology by SEM

**Figure 5:**
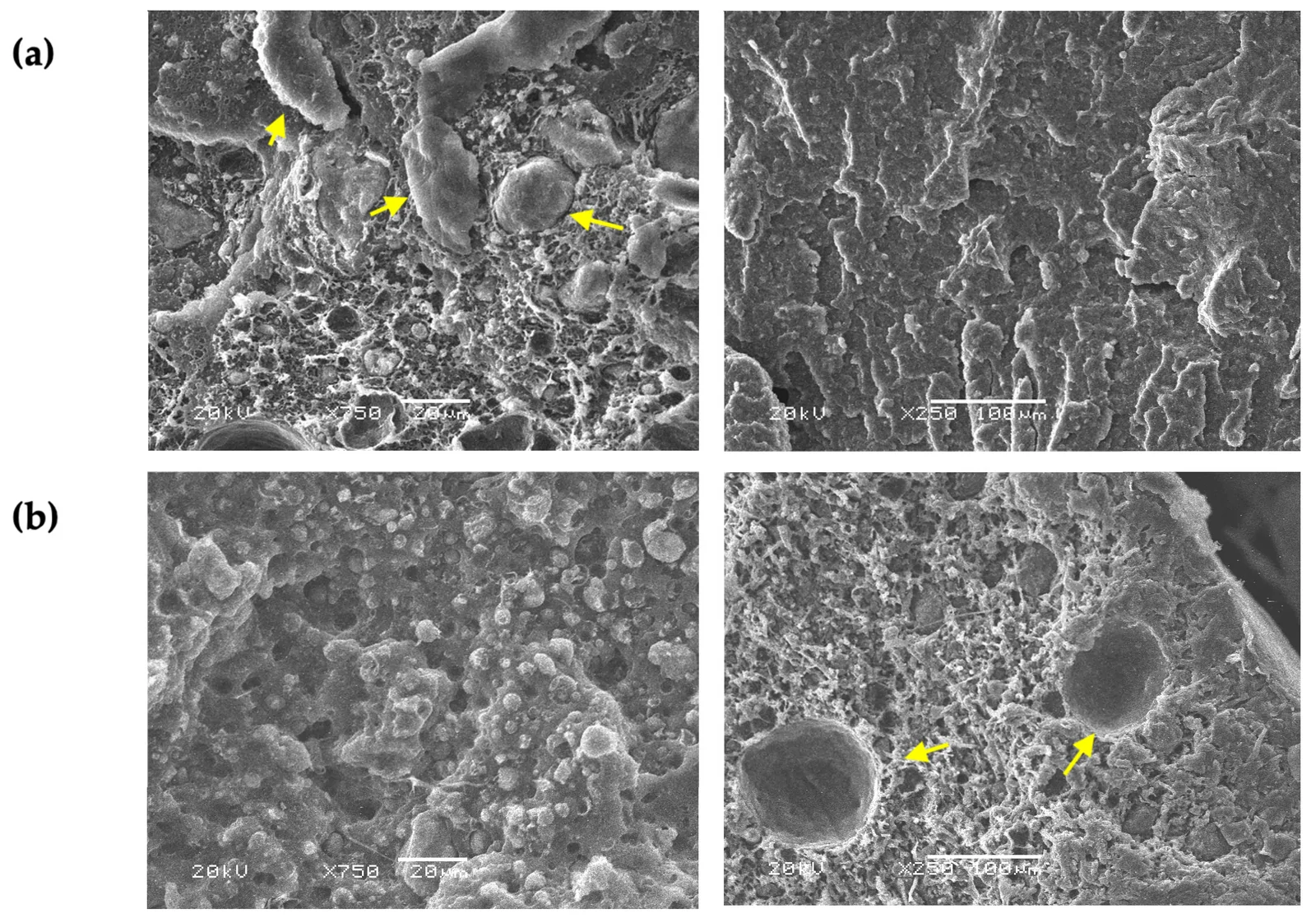
SEM micrographs of fracture surface morphology of the PCL/PLA/α-TCP wt.20%/ZrO_2_ wt.5% composite filaments fabricated using the Noztek Touch (**a**) and Brabender Plasti-Corder (**b**) extrusion systems at a magnification of ×250 and ×750. Yellow arrows indicate representative particle agglomerates and pores.

SEM micrographs of the cryo-fractured surfaces (Figure 5) revealed that both composite filaments exhibited irregular surface morphologies, reflecting the heterogeneous incorporation of the ceramic phases within the PLA/PCL polymer matrix. The presence of rough regions, dispersed particulate structures, and surface irregularities is consistent with the incorporation of α-TCP and ZrO_2_ particles into the polymer matrix. Similar surface features have been reported for biodegradable polymer-ceramic composites containing calcium phosphate fillers, where increasing the ceramic content contributes to higher surface roughness and porosity [34].

Clear differences in surface morphology were observed between the two processing routes. The filament processed using the Noztek Touch exhibited localized particle agglomerates (Figure 5a, arrows), suggesting a less homogeneous dispersion of the ceramic fillers throughout the polymer matrix. In contrast, the filament processed using the Brabender Plasti-Corder presented a more uniform particle distribution, although larger pores were observed on the fracture surface (Figure 5b, arrows). Quantitative measurements of the observed morphological features showed that particle/agglomerate areas ranged from 14.18 to 631.92 μm^2^ for the Noztek processing route, while they varied from 7.70 to 103.41 μm^2^ for the Brabender Plasti-Corder system. In contrast, pore areas ranged from approximately 19.97 to 81.48 μm^2^ in the Noztek samples, whereas substantially larger pore areas, ranging from 357.25 to 11,135.78 μm^2^, were observed in the Brabender samples. The formation of these pores may be attributed to residual moisture, entrapped air during melt processing, or differences in the interfacial compatibility between the polymeric matrix and ceramic fillers, which can promote localized void formation during extrusion. Previous studies have also reported that zirconia particles exhibit limited interfacial interactions with polyester matrices, favoring the formation of interfacial voids when filler dispersion is not optimal [35].

These morphological differences may influence the performance of the composite biomaterials. Particle agglomerates can act as stress concentration sites that facilitate crack initiation and propagation under mechanical loading, whereas excessive porosity may reduce its mechanical performance [36]. Recent studies on 3D-printed PLA/PCL composites have reported that variations in ceramic filler content can affect particle distribution and pore morphology, with consequent effects on mechanical performance [12]. Conversely, from a biological perspective, the surface roughness observed in both composite filaments may be advantageous for cell-material interactions, since moderately rough surfaces have been associated with enhanced cell adhesion, proliferation, and extracellular matrix deposition [37]. Together with the FTIR, Raman spectroscopy, and XPS analyses, the SEM observations indicate that although both extrusion methods preserved the chemical composition with no evident polyester degradation of the composite, they generated distinct surface morphologies that may influence its subsequent mechanical and biological performance.

### 3.3. Mechanical Properties Under Compression

#### Compressive Test

**Figure 6:**
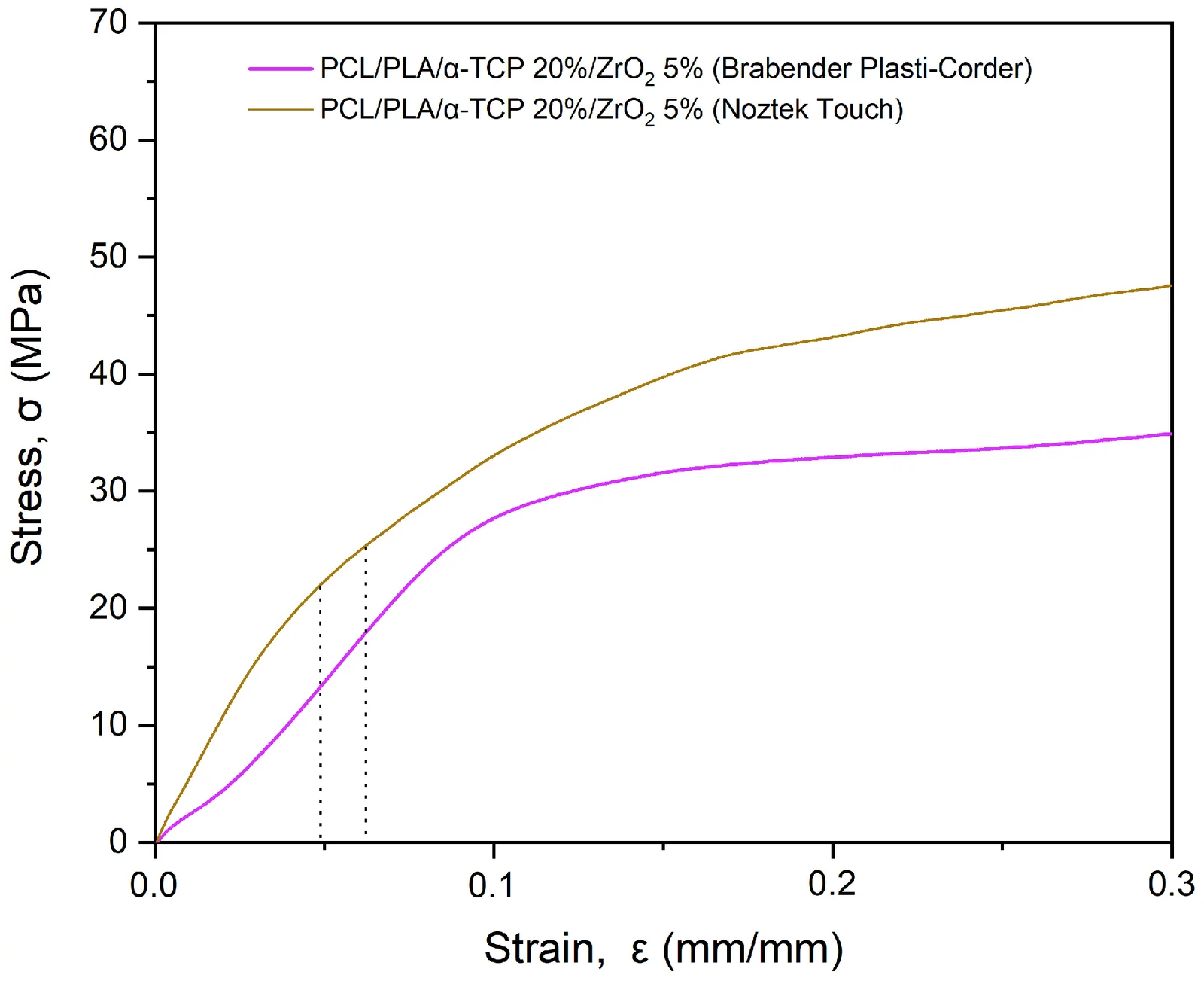
Stress–strain curves of PCL/PLA/α-TCP 20%/ZrO_2_ 5% fabricated from Noztek Touch and the Brabender Plasti-Corder.

Figure 6 shows a representative stress–strain curve from the compression testing of composite biomaterial filaments (PCL/PLA/α-TCP 20%/ZrO_2_ 5%) fabricated from Noztek and the Brabender system. The Young’s modulus was determined from the slope between two points, 0.04–0.06 mm/mm, in the linear elastic region of the mechanical response.

Compression testing showed a numerical difference in the mechanical response of the composite filaments produced using the two fabrication methods. The Noztek extruded filaments exhibited a higher elastic modulus (258.44 ± 73 MPa) than those filaments produced using the Brabender Plasti-Corder system (155.98 ± 113 MPa). Both biomaterials showed a typical ductile response under compressive loading. Previous studies on polymeric composites reinforced with calcium phosphates have shown that the incorporation of ceramic particles can modify the stiffness and mechanical performance of the polymeric matrix depending on filler concentration, particle distribution, and interfacial adhesion between phases [30,38].

ZrO_2_ has been reported as a reinforcing phase capable of improving the mechanical behavior of polymer-based biomaterials, although its effect strongly depends on particle dispersion and processing conditions [39]. The lower mean elastic modulus observed in the Brabender filaments may be associated with the internal porosity identified by SEM analysis. These pores can act as stress concentrators and negatively affect the mechanical performance of porous polymeric structures [40]. However, this interpretation should be considered a possible contributing factor rather than a demonstrated cause, as the difference between processing routes was not statistically significant (*p* = 0.140). These findings suggest that, although both processing methods produced the same chemical composition, differences in the resulting microstructure may have contributed to the observed variation in the mechanical response of the composite biomaterial.

The compressive elastic modulus obtained in this study ranged from 155.98 MPa for the Brabender-extruded filaments to 258.44 MPa for the Noztek-extruded filaments. These values are lower than those reported for conventional glass fiber posts, which are mainly designed for permanent dentition and require higher stiffness to withstand long-term functional loads [41]. However, for a biodegradable dental post intended for primary teeth, a lower stiffness may be advantageous because it could provide a more compliant mechanical response and potentially reduce stress concentration at the tooth-post interface [42]. Considering the temporary nature of primary teeth and the need for a resorbable alternative, the mechanical requirements of this type of biomaterial may differ from those of permanent endodontic posts. Therefore, the compressive mechanical performance obtained represents an initial approach toward the development of a biodegradable pediatric endodontic post, although further optimization of the composite formulation and processing conditions may be necessary to enhance its mechanical stability.

### 3.4. Accelerated Degradation

The mass loss of the composite filament obtained by Brabender extrusion and pristine PLA and PCL polymers after degradation in different media is summarized in Table 3. Under reflux conditions in distilled water, all evaluated biomaterials exhibited degradation percentages below 1%, indicating high hydrolytic stability under these conditions. The limited mass loss suggests that even when hydrolysis of ester bonds is possible, there is a need for a catalyst; therefore, no significant structural deterioration of the composite biomaterials was observed [43,44].

In an acidic medium (HCl 2N), the composite material showed 2.1% mass loss, which was lower than that of pristine PCL (100%) and PLA (5.3%). This suggests a possible protective or neutralizing effect associated with the presence of ceramic phases. This behavior may be related to the incorporation of ceramic fillers, since previous studies have shown that calcium phosphate-based materials can influence degradation kinetics and contribute to buffering local pH changes, reducing the susceptibility of polymeric matrices to acidic hydrolysis [45].

In the case of H_2_O_2_, limited mass loss was also observed. These results suggest that, under the evaluated conditions, the induced oxidative stress was not sufficient to promote significant structural changes in the polymeric matrix, indicating relatively favorable stability against short-term oxidation. However, previous studies have reported that oxidative environments may promote chain scission reactions in PLA, although the extent of degradation strongly depends on exposure conditions, including oxidant concentration and treatment time [46].

**Figure 7:**
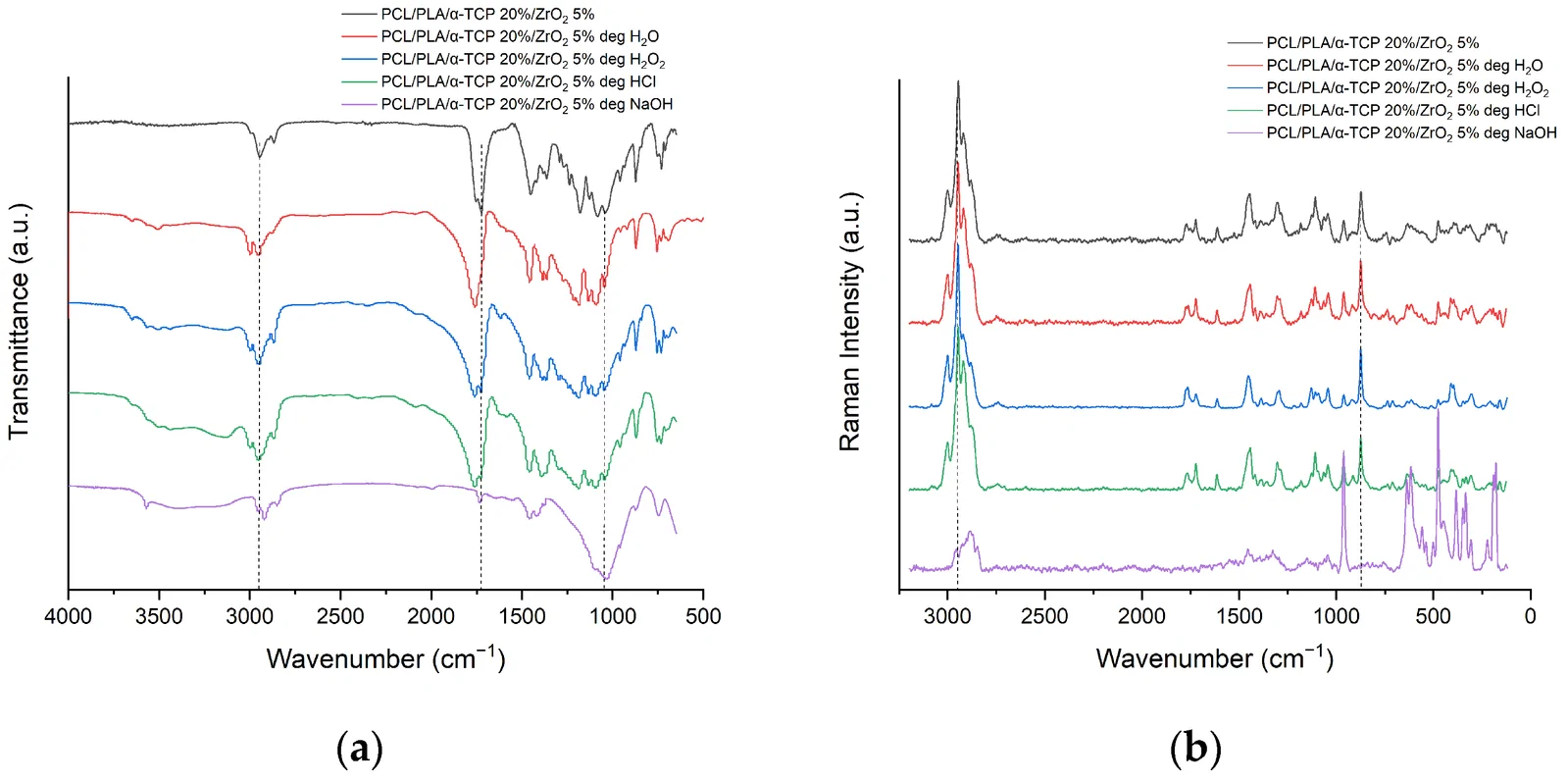
FTIR (**a**) and Raman (**b**) spectra of PCL/PLA-based composites before and after accelerated degradation under different chemical conditions.

The analysis of the samples exposed to NaOH 5N provided the clearest evidence of chemical degradation of the material. The results indicate that alkaline hydrolysis was the predominant degradation mechanism, where the filler had some buffering effect, as degradation was lower than in pristine polymers. This was confirmed by FTIR analysis (see Figure 7a), where a decrease in the band associated with C=O stretching, which is fundamental in the structure of both PCL and PLA, was observed. However, bands located around 1000–500 cm^−1^, associated with phosphate groups and ZrO_2_ vibrations, remained. In the NaOH 5N-treated sample, as most of the polymer matrix is removed, these ceramic-related bands become more pronounced and defined. In the Raman spectra, the intensity of the peaks decreases, while new prominent peaks appear in the low wavenumber region. These changes are consistent with degradation of the polymeric matrix and the exposure of inorganic phases that remain after a mass loss of approximately 68% of the polymer fraction. Previous reports using FTIR analysis on PLA subjected to chemical degradation have described similar behavior, showing a reduction in carbonyl bands together with an increase in signals related to hydrolysis-derived products, confirming progressive changes in the chemical structure of the polymer during the degradation process [47].

Raman and FTIR spectra (Figure 7) revealed minimal structural changes in samples exposed to distilled water, HCl 2N, and H_2_O_2_ media, where the characteristic bands of the polymeric matrix remained largely preserved, indicating limited degradation. In contrast, samples exposed to NaOH showed the most significant spectral changes.

### 3.5. Biological Studies

#### 3.5.1. Biocompatibility Test

**Figure 8:**
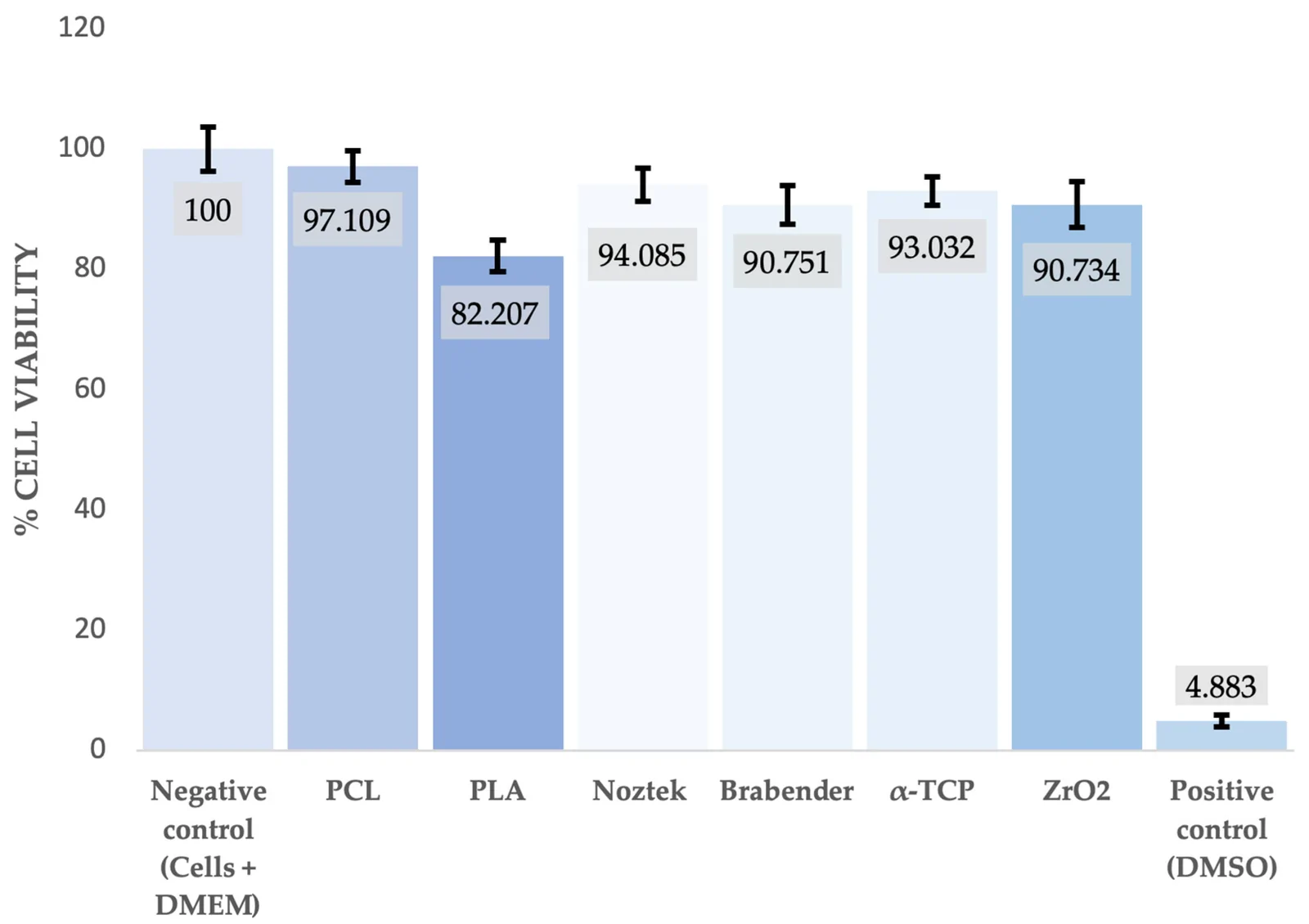
Cell viability of hDPSCs after 24 h of incubation with the individual components and composite biomaterial, as determined by the MTT assay. Data are presented as mean + SD (n = 10), corresponding to two independent experiments with five technical replicates per material in each experiment.

All composites and individual components exhibited cell viability values higher than 80% (Figure 8), indicating that none of the tested materials induced cytotoxic effects. According to ISO 10993-5 [48], cell viability values above 70% classify a material as non-cytotoxic, confirming the excellent cytocompatibility of all developed biomaterials. PCL showed the highest viability (97.1%), which is consistent with its well-documented biocompatibility and widespread use in tissue engineering applications [49]. The composite biomaterial filaments fabricated by Noztek (94.1%) and Brabender Plasti-Corder (90.8%) also exhibited high cell viability, indicating that the incorporation of the ceramic phases did not adversely affect cellular response.

The presence of α-TCP may contribute to a favorable biological environment due to its bioactive nature and similarity to the mineral phase of hard tissues [50]. In addition, ZrO_2_ has been widely used in biomedical and dental applications because of its biocompatibility, chemical stability, and favorable cellular interactions. Previous studies have reported that PCL/ZrO_2_ hybrid materials support higher cell viability than ZrO_2_ alone, suggesting that the combination of polymeric and ceramic phases may enhance cellular response [51].

Although PLA showed the lowest viability among the tested materials (82.2%), the value remained well above the cytotoxicity threshold and agrees with previous studies reporting good cytocompatibility of PLA-based biomaterials [52]. These findings suggest that the extrusion process and the incorporation of ceramic fillers did not compromise the biocompatibility of the developed composite biomaterial.

Overall, these findings demonstrate that neither the incorporation of α-TCP and ZrO_2_ nor the extrusion process compromised the cytocompatibility of the composite biomaterials. Moreover, the comparable biological response observed for the Noztek and Brabender filaments indicates that both processing methods are suitable for producing biomaterials intended for dental tissue engineering applications.

Statistical analysis was performed using one-way analysis of variance (ANOVA) to compare cell viability among the evaluated groups. Significant differences were observed (*p* < 0.001). Tukey’s multiple comparison test (95% confidence level) showed that all biomaterials and their individual components did not differ significantly from the negative control, indicating the absence of cytotoxicity. In contrast, the positive control (DMSO) showed significantly lower cell viability than all other groups (*p* < 0.05).

#### 3.5.2. Evaluation of Cell Adhesion by CellTracker^TM^ Green CMFDA

**Figure 9:**
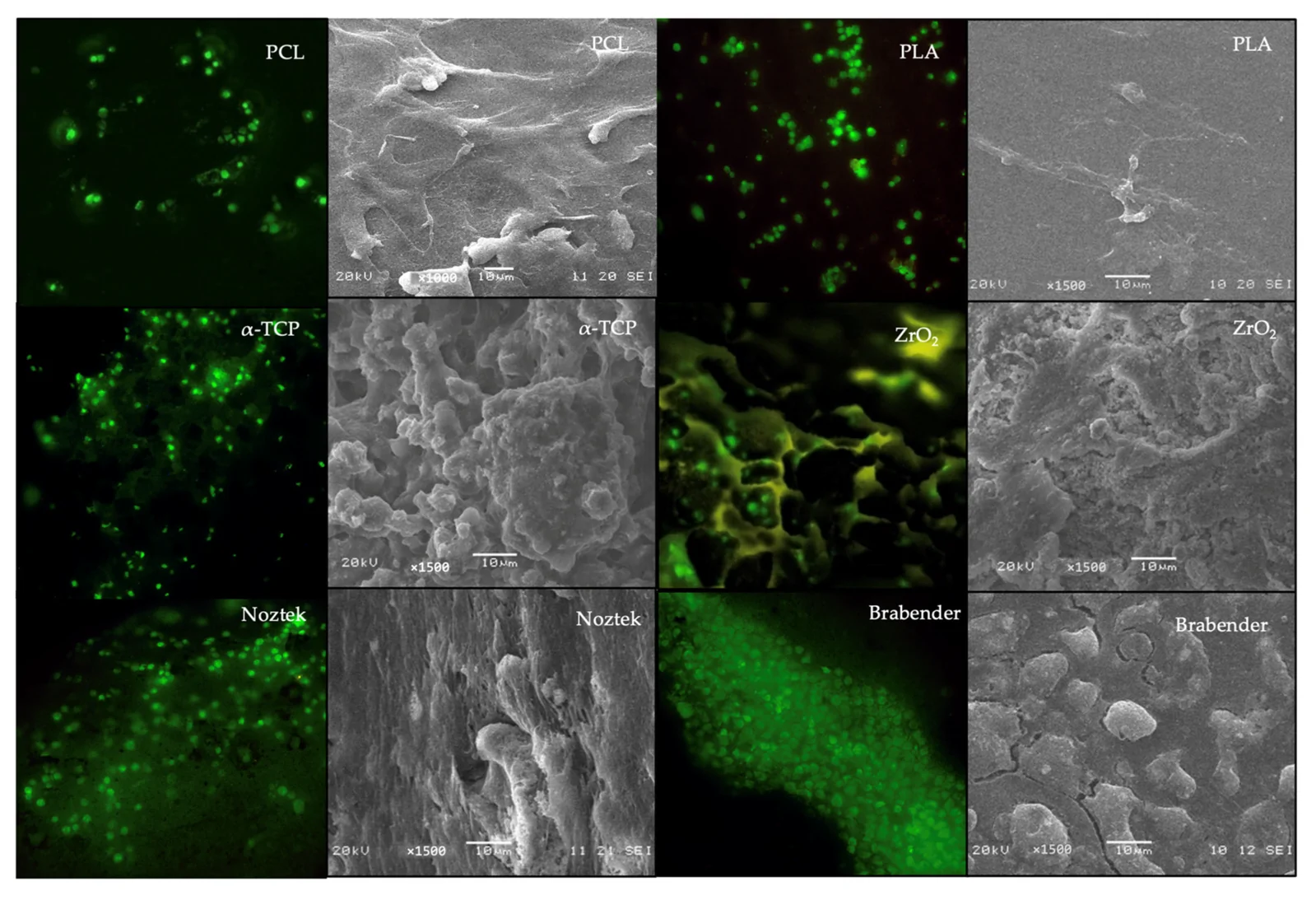
Fluorescence micrographs and SEM images of the interaction of HDPSCs with individual components and composite biomaterial labeled with CellTracker™ Green after 24 h of culture.

Fluorescence microscopy and SEM observations (Figure 9) demonstrate successful adhesion and homogeneous distribution of human dental pulp stem cells (hDPSCs) on all evaluated biomaterials after 24 h of culture. CellTracker^TM^ Green fluorescence confirmed the presence of viable adherent cells on both the individual components and the composite biomaterials. The observed cell distribution across the biomaterial surfaces indicates that the developed composites provide a favorable substrate for early cell attachment and cell-material interactions.

SEM analysis revealed differences in the surface morphology of the composite biomaterials after extrusion. Both processing methods generated surfaces with heterogeneous topography and microscale irregularities, which may have favored early cell attachment by increasing the available surface area and providing additional anchoring sites for cell-material interactions. The observed cellular distribution is consistent with previous studies demonstrating that PCL-based biomaterials support the adhesion and spreading of human dental pulp stem cells during the early stages of culture [53].

Furthermore, the incorporation of α-TCP and ZrO_2_ did not prevent cell attachment to the composite biomaterial surface. Therefore, the composite PCL, PLA, α-TCP, and ZrO_2_ provides a suitable substrate for the initial adhesion of hDPSCs. These findings, together with the MTT results, support the potential of the developed biomaterial as a candidate for dental tissue engineering and regenerative applications.

Collectively, the MTT assay, fluorescence microscopy, and SEM observations demonstrate that the developed composite biomaterials exhibit excellent cytocompatibility, support early cell adhesion, and maintain favorable cell-material interactions irrespective of the extrusion method employed. These findings reinforce the potential of the developed composite as a biodegradable dental post material for regenerative pediatric dentistry.

#### 3.5.3. Antimicrobial Activity

The agar diffusion assay revealed no inhibition zones against either *Staphylococcus aureus* ATCC 25923 or *Escherichia coli* ATCC 25922 for the composite biomaterial (Table 4). In contrast, the positive controls produced well-defined inhibition halos, whereas no inhibition was observed around the negative controls, confirming the validity of the assay. These results indicate that, under the experimental conditions evaluated, the developed composite biomaterial did not exhibit detectable antibacterial activity from diffusible agents against either bacterial strain.

Previous studies have reported antibacterial activity of ZrO_2_ nanoparticles against both Gram-positive and Gram-negative bacteria, which has been attributed to the generation of reactive oxygen species (ROS), oxidative stress, and subsequent disruption of bacterial cell membranes [20]. Likewise, antibacterial effects have been described in ZrO_2_-containing polymeric nanocomposites and dental coatings, where inhibition zones against *Staphylococcus aureus*, *Escherichia coli*, and other oral pathogens have been observed [54,55].

In contrast to these reports, no antibacterial activity was detected in the present study for pristine ZrO_2_ particles. These differences may be associated with several factors, including zirconia particle size, concentration, dispersion within the polymer matrix, and surface exposure of the ceramic phase. In the present work, ZrO_2_ was incorporated at 5 wt.%, and XPS analysis did not detect zirconium on the outermost surface of the composite biomaterials, suggesting that the ceramic particles were largely covered by the PLA/PCL matrix. Such surface coverage may have limited the direct interaction between zirconia particles and bacterial cells, potentially contributing to the lack of detectable antibacterial activity.

Furthermore, the agar diffusion assay primarily detects antimicrobial compounds capable of diffusing through the culture medium. Therefore, the absence of inhibition zones does not necessarily exclude the possibility of contact-dependent antibacterial effects, particularly in polymeric composite biomaterials where the ceramic particles remain embedded within the matrix. Additional studies using direct-contact antibacterial assays or CFU-based assays would provide a more comprehensive evaluation of the antimicrobial performance of the developed composites.

Overall, although the incorporation of ZrO_2_ did not confer detectable antibacterial activity under the conditions evaluated, the extrusion process successfully preserved the chemical stability, cytocompatibility, and structural integrity of the composite biomaterials, as demonstrated by the FTIR, Raman, XPS, SEM, and cell viability analyses. Therefore, the absence of antibacterial activity under these conditions does not diminish the potential of these biomaterials for use as biodegradable dental posts for primary teeth, where mechanical performance, controlled biodegradation, and biocompatibility constitute the primary design requirements.

### 3.6. Prototype of Dental Post by 3D Printing

#### Preliminary Development of a Dental Post by 3D Printing

**Figure 10:**
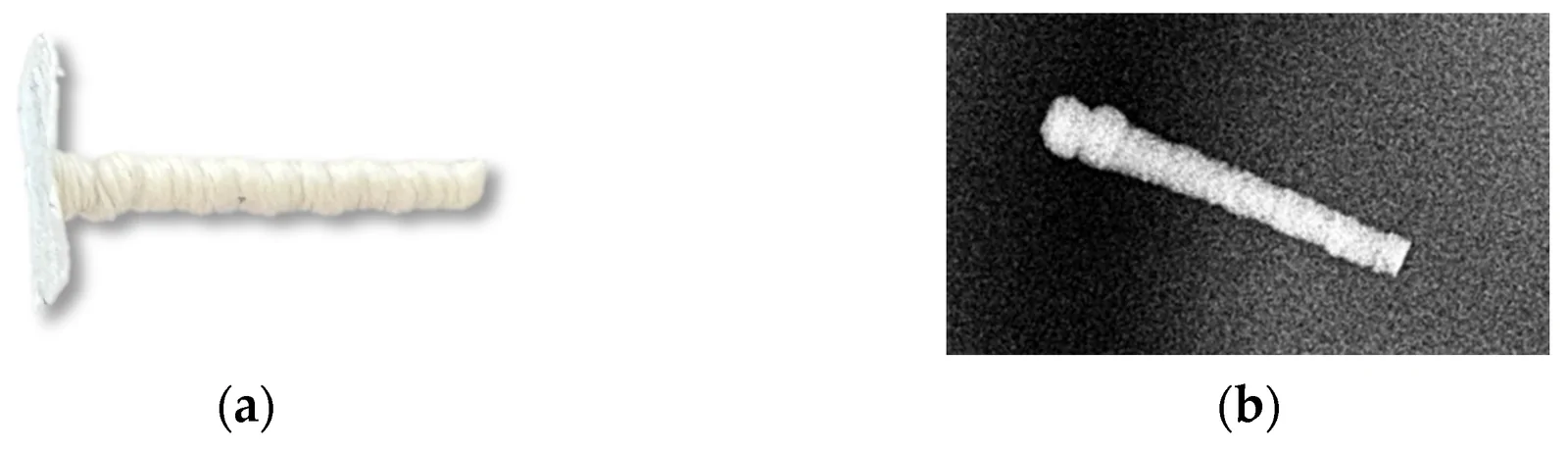
Preliminary 3D-printed dental post fabricated from the PCL/PLA/α-TCP 20%/ZrO_2_ 5% biocomposite filament (**a**) and qualitative radiographic image (**b**) of the printed prototype demonstrating its radiographic detectability.

The biomaterial exhibited adequate extrudability and shape retention during the 3D printing process, as shown in Figure 10. These characteristics are essential for the development of customized endodontic posts, as consistent filament formation and dimension stability are required to obtain structures with reproducible geometry during extrusion-based manufacturing processes [56].

The successful printing of the PCL/PLA-based composite reinforced with α-TCP and ZrO_2_ suggests that the incorporation of the ceramic phases did not compromise the processability observed during extrusion, allowing the production of filaments suitable for further processing and potential fabrication of resorbable posts in primary teeth. The preliminary 3D-printed post exhibited a compressive elastic modulus of 109 + 11 MPa, providing an initial indication of its mechanical response after the printing process.

A qualitative radiographic image of the preliminary 3D-printed post demonstrated that the prototype was radiographically visible. This observation is attributable to the incorporation of ZrO_2_, which is commonly used as a radiopacifying agent in dental materials, although α-TCP may also contribute. Since radiopacity was not quantitatively assessed, further studies are required to determine its radiographic performance.

## 4. Conclusions

This study demonstrated the successful development of composite biomaterial filaments based on polylactic acid (PLA), polycaprolactone (PCL), α-tricalcium phosphate (α-TCP), and zirconia (ZrO_2_), using two different processing routes. FTIR and Raman analyses revealed no thermal degradation of polymeric matrices and confirmed the preservation of the characteristic chemical structure of both polymers, as well as the presence of the incorporated ceramic components, indicating that neither processing route induced significant chemical modifications in the developed composite.

Accelerated biodegradation showed weight loss behavior consistent with biodegradable polymer systems, allowing the establishment of a preliminary degradation profile under different chemical environments, i.e., in the presence of acidophilus bacteria or in contact with Vitapex/Ultrapex of an alkaline nature. In addition, the material exhibited favorable physicochemical properties, while the incorporation of zirconia may contribute to radiopacity, an essential characteristic for radiographic monitoring in dental applications.

Biological evaluation demonstrated that the composite did not induce cytotoxic effects and supported initial cell attachment, suggesting adequate biocompatibility for potential dental and biomedical applications.

Overall, these preliminary findings identify the PCL/PLA/α-TCP 20%/ZrO_2_ 5% composite as a promising candidate material for additive manufacturing and for the development of potentially resorbable intracanal posts for primary teeth. However, further studies are required to establish its degradation behavior under physiologically relevant conditions, its relationship with physiological root resorption, and its effect on the permanent successor teeth. Additional mechanical (3- or 4-point bending, the determination of Kic or Gic for fracture toughness and fatigue behavior) and tooth-post system evaluations are also necessary to establish its suitability for dental applications after incorporating an effective antimicrobial agent. The developed composite may provide an alternative approach to conventional materials by offering temporary mechanical support, a favorable biological response, and adequate radiopacity for clinical evaluation.

## Figures and Tables

**Table 1:** Composition and batch sizes of the PLA/PCL-based composite biomaterial prepared using the two processing routes.

Composite	PCL/PLA/α-TCP 20%/ZrO_2_ 5% (Noztek Touch)	PCL/PLA/α-TCP 20%/ZrO_2_ 5% (Brabender Plasti-Corder)
PCL	22.5 g	37.5 g
PLA	22.5 g	37.5 g
α-TCP 20%	12 g	20 g
ZrO_2_ 5%	3 g	5 g

**Table 2:** Atomic percentage of C, O, Ca, P, and Zr of filaments composed of PCL/PLA/α-TCP 20%/ZrO_2_ 5%.

%	PCL + PLA + α-TCP 20% + ZrO_2_ 5% (Noztek)	PCL + PLA + α-TCP 20% + ZrO_2_ 5% (Brabender Plasti-Corder)
C	85.35	81.93
O	13.05	13.08
Ca	1.6	3.72
P	ND	ND
Zr	ND	ND

ND: Not detected.

**Table 3:** Mass loss (%) of composite and pristine polymers after accelerated degradation in different chemical media.

Material	H_2_O (%)	HCl 2N (%)	NaOH 5N (%)	H_2_O_2_ (%)
PCL	0.87 ± 0.90	100.00	100.00	0.59 ± 0.19
PLA	0.47 ± 0.11	5.33 ± 0.41	96.49 ± 0.10	2.80 ± 1.54
PCL/PLA/α-TCP 20%/ZrO_2_ 5% (Brabender)	0.93 + 0.40	2.10 ± 0.032	68.68 ± 1.64	2.95 ± 1.47

**Table 4:** Antimicrobial activity, with *Staphylococcus aureus* and *Escherichia coli* incubated for 16–18 h at 35 °C for PCL/PLA/α-TCP/ZrO_2_ composites obtained by Brabender extrusion.

	Biomaterial PCL/PLA/α-TCP 20%/ZrO_2_ 5%	Control (+)	Control (−)
*Staphylococcus* *aureus* *ATCC 25923*	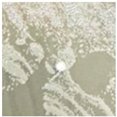	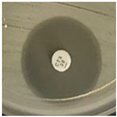	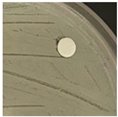
*Escherichia coli* *ATCC 25922*	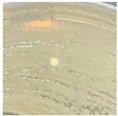	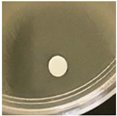	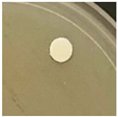

## Data Availability

The data presented in this study are available on request from the corresponding author. The data are not publicly available because their content is part of a PhD program in Health Sciences.

## Abbreviations

The following abbreviations are used in this manuscript:

PCL	Polycaprolactone
PLA	Polylactic Acid
α-TCP	Alpha Tricalcium Phosphate
ZrO_2_	Zirconium Oxide
